# A User-Friendly, Web-Based Integrative Tool (ESurv) for Survival Analysis: Development and Validation Study

**DOI:** 10.2196/16084

**Published:** 2020-05-05

**Authors:** Kyoungjune Pak, Sae-Ock Oh, Tae Sik Goh, Hye Jin Heo, Myoung-Eun Han, Dae Cheon Jeong, Chi-Seung Lee, Hokeun Sun, Junho Kang, Suji Choi, Soohwan Lee, Eun Jung Kwon, Ji Wan Kang, Yun Hak Kim

**Affiliations:** 1 Department of Nuclear Medicine Pusan National University Hospital Busan Republic of Korea; 2 Department of Anatomy, School of Medicine Pusan National University Yangsan Republic of Korea; 3 Department of Orthopaedic Surgery Pusan National University Hospital Busan Republic of Korea; 4 Deloitte Analytics Group Deloitte Consulting LLC Seoul Republic of Korea; 5 Biomedical Research Institute Pusan National University Hospital Busan Republic of Korea; 6 Department of Biomedical Engineering, School of Medicine Pusan National University Busan Republic of Korea; 7 Department of Statistics Pusan National University Busan Republic of Korea; 8 Department of Biomedical Informatics, School of Medicine Pusan National University Yangsan Republic of Korea

**Keywords:** survival analysis, grouped variable selection, The Cancer Genome Atlas, web-based tool, user service

## Abstract

**Background:**

Prognostic genes or gene signatures have been widely used to predict patient survival and aid in making decisions pertaining to therapeutic actions. Although some web-based survival analysis tools have been developed, they have several limitations.

**Objective:**

Taking these limitations into account, we developed ESurv (Easy, Effective, and Excellent Survival analysis tool), a web-based tool that can perform advanced survival analyses using user-derived data or data from The Cancer Genome Atlas (TCGA). Users can conduct univariate analyses and grouped variable selections using multiomics data from TCGA.

**Methods:**

We used R to code survival analyses based on multiomics data from TCGA. To perform these analyses, we excluded patients and genes that had insufficient information. Clinical variables were classified as 0 and 1 when there were two categories (for example, chemotherapy: no or yes), and dummy variables were used where features had 3 or more outcomes (for example, with respect to laterality: right, left, or bilateral).

**Results:**

Through univariate analyses, ESurv can identify the prognostic significance for single genes using the survival curve (median or optimal cutoff), area under the curve (AUC) with C statistics, and receiver operating characteristics (ROC). Users can obtain prognostic variable signatures based on multiomics data from clinical variables or grouped variable selections (lasso, elastic net regularization, and network-regularized high-dimensional Cox-regression) and select the same outputs as above. In addition, users can create custom gene signatures for specific cancers using various genes of interest. One of the most important functions of ESurv is that users can perform all survival analyses using their own data.

**Conclusions:**

Using advanced statistical techniques suitable for high-dimensional data, including genetic data, and integrated survival analysis, ESurv overcomes the limitations of previous web-based tools and will help biomedical researchers easily perform complex survival analyses.

## Introduction

The accumulation of large amounts of genomic data following the development of next-generation sequencing techniques is paving the way toward precision medicine [1-4]. In particular, gene expression profiles or signatures have been widely used to predict patient prognosis and assist in deciding therapeutic strategies for the treatment of various cancers [2,5-9].

Genomic data sets are highly variable, with that variability rising with an increasing number of patients, making it high-dimensional in nature. To efficiently link high-dimensional genomic and survival data, statisticians have developed grouped variable selection models, based on the Cox proportional hazards model, including the following: least absolute shrinkage and selection operator (lasso), elastic net regularization (elastic net), and network-regularized high-dimensional Cox-regression (Coxnet, hereon referred to as Net) [2,10-13]. Among these methods, Net has been found to have the fewest overfitting problems and the highest prediction performance in these applications, as it takes into consideration the complexities of biological networks [2,6,8,10,14].

Successfully identifying and verifying prognostic factors using big databases is essential in medical research, but this can be difficult for researchers who are unfamiliar with computer science. To address this unmet clinical need, some web-based survival analysis tools have been developed. Although these tools have some limitations, they have been used in some univariate analyses [15-18]. SurvExpress [19], PROGgene [20], and PrognoScan [21] are popular web-based survival analysis tools that calculate the statistical significance of a prognosis using only messenger RNA (mRNA) expression data [15-18]. The limitations of previous tools include the following: (1) The use of mRNA expression as a simple categorical value to provide Kaplan‒Meier curves for all patients, regardless of their characteristics. The use of a continuous variable like mRNA as a categorical factor can change the nature of the variable arbitrarily, resulting in serious errors. (2) These tools do not provide gene/variable signatures that are statistically better, in terms of predicting prognosis, than a single gene. (3) They do not consider cancer classifications like histological type. (4) Users cannot use their own data. (5) These tools do not provide high-quality images and tabular results. (6) Users cannot create a risk-scoring system by specifying the genes of interest.

To overcome these limitations, we developed ESurv (Easy, Effective, and Excellent Survival analysis tool [22]), which is an online web resource for identifying prognostic biomarkers in pan-cancer from The Cancer Genome Atlas (TCGA) or user data.

## Methods

### Processing Genetic and Clinical Information From Patients

We performed survival analyses based on multiomics and clinical data from TCGA (Table 1), obtained via Broad GDAC Firebrowse [23] and the GDC Data Portal [24-26]. We used level 3 RNAseq (RNA sequencing), miRNAseq (microRNA sequencing), and methylation array data. The criteria for exclusion were as follows: genes with 0 values of more than 10% (when the amount of missing data is greater than 10%, the results may be biased) [27], patients with insufficient overall survival information (survival time or status), and patients with paired normal tissue and metastasis samples in TCGA.

**Table 1:** Summary of the data available in the ESurv.

Cancers with available omics data	Messenger RNA (Yes/No)	MicroRNA (Yes/No)	Methylation (Yes/No)	Total patients, n
Acute myeloid leukemia	Yes	Yes	Yes	200
Adrenocortical carcinoma	Yes	Yes	Yes	92
Bladder urothelial carcinoma	Yes	Yes	Yes	412
Brain lower grade glioma	Yes	Yes	Yes	515
Breast invasive carcinoma	Yes	Yes	Yes	1097
Cervical and endocervical carcinoma	Yes	Yes	Yes	307
Cholangiocarcinoma	Yes	Yes	Yes	45
Colon adenocarcinoma	Yes	Yes	Yes	458
Esophageal carcinoma	Yes	Yes	Yes	185
Glioblastoma multiforme	Yes	No	Yes	595
Head and neck squamous cell carcinoma	Yes	Yes	Yes	528
Kidney chromophobe	Yes	Yes	Yes	113
Kidney renal clear cell carcinoma	Yes	Yes	Yes	537
Kidney renal papillary cell carcinoma	Yes	Yes	Yes	291
Liver hepatocellular carcinoma	Yes	Yes	Yes	377
Lung adenocarcinoma	Yes	Yes	Yes	522
Lung squamous cell carcinoma	Yes	Yes	Yes	504
Lymphoid neoplasm diffuse large B cell lymphoma	Yes	Yes	Yes	48
Mesothelioma	Yes	Yes	Yes	87
Ovarian serous cystadenocarcinoma	Yes	Yes	No	591
Pancreatic adenocarcinoma	Yes	Yes	Yes	185
Pheochromocytoma and paraganglioma	Yes	Yes	Yes	179
Prostate adenocarcinoma	Yes	Yes	Yes	499
Rectum adenocarcinoma	Yes	Yes	Yes	171
Sarcoma	Yes	Yes	Yes	261
Skin cutaneous melanoma	Yes	Yes	Yes	470
Stomach adenocarcinoma	Yes	Yes	Yes	443
Testicular germ cell tumor	Yes	Yes	Yes	134
Thymoma	Yes	Yes	Yes	124
Thyroid carcinoma	Yes	Yes	Yes	516
Uterine carcinosarcoma	Yes	Yes	Yes	57
Uterine corpus endometrial carcinoma	Yes	Yes	Yes	548
Uveal melanoma	Yes	Yes	No	80

### Processing Clinical Variables in Net

Clinical variables (tumor stage, age, sex, cancer type, blast count, histologic grade, laterality, anatomic neoplasm subdivision, tumor tissue site, and human papillomavirus status) can be included in the Net depending on cancer variety, allowing for sophisticated analyses. Clinical variables were classified as 0 and 1 when there were two categories (for example, chemotherapy: no or yes), and dummy variables were applied when the clinical variable could fall into three or more categories (for example, laterality: right, left, or bilateral).

### Grouped Variable Selections for Creating Gene/Variable Signatures

ESurv uses one of the following three methods: least absolute shrinkage and selection operator (lasso), elastic net regularization (elastic net), and network-regularized high-dimensional Cox-regression (Net) using the Coxnet package (version 0.2) in R [2,10,12,14]. In Net analysis, we transformed the topological pathway information of large databases (KEGG [Kyoto Encyclopedia of Genes and Genomes], Biocarta, HumanCyc, Reactome, Panther, and NCI [National Cancer Institute]) into a gene network matrix using the graphite package (version 3.10) in R. Users can set the mixing parameter alpha, which decides the balance between lasso and ridge regression [10,14]. All grouped variable selections use 10-fold cross-validation. After variable selection, we calculated the prognostic score by multiplying the variable value by the regression coefficient.

### Statistical Analysis

To determine the optimal cutoff value, preventing overoptimization, we used the maximal UNO’s C-index and a 5-fold cross validation. For the Kaplan-Meier survival curves, patients were divided into two groups, high- and low-risk, based on specific gene expression parameters (median cutoff or optimal cutoff value), with a *P* value determined by a log-rank test. The C-index and area under the curve (AUC) value were used to evaluate the effect of specific variables on survival [28]. These results can be seen not only in all patients but also in patient subgroups based on sex or stage. The results were obtained using R packages survival (version 2.44-1.1), survMisc (version 0.5.5), coin (version 1.3-0), MASS (Modern Applied Statistics with S, version 7.3-51.4), edgeR (version 3.24.3), and survAUC (version 1.0-5). All graphical outputs from ESurv were plotted using the plotly (version 4.9.0) R package. All data cleaning and statistical analyses in this study were performed using R statistical software (version 3.6.0, R Core Team, R Foundation for Statistical Computing).

### Implementation

The ESurv web server implements AngularJS with HTML5 to display analyzed data through a web query interface. The results of these analyses were calculated on demand on a backend server running Java Servlet in conjunction with the R statistical program.

## Results

### Running ESurv

**Figure 1:**
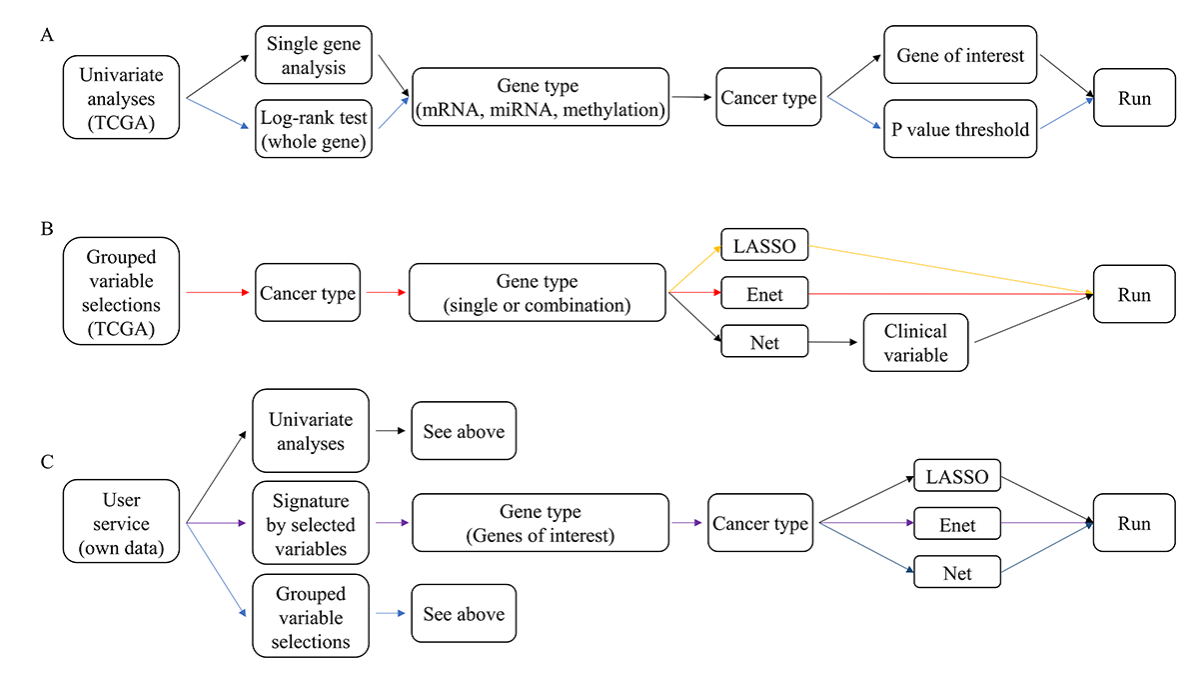
The running procedure of ESurv.

Details of the running procedure for ESurv are described in Figure 1. First, users can choose one of three methods: univariate survival analyses, grouped variable selections, or user service.

After choosing univariate survival analysis, users can select single gene analysis or log-rank test of whole genomes. In single gene analysis, users select the type of cancer, gene data type (mRNA, microRNA [miRNA], or methylation), gene of interest, and time for the receiver operating characteristics (ROC) curve, in that order. For log-rank testing of whole genomes, users choose the type of cancer, gene data type (mRNA, miRNA, or methylation), time for the ROC curve, and *P* value threshold, in that order.

If users instead choose grouped variable selections, they must select the type of cancer, gene data (mRNA, miRNA, methylation, or integrative analysis), grouped variable selection method (lasso, elastic net, or Net), time for the ROC curve, and alpha, in that order. Alpha decides the balance between ridge and lasso penalties; the larger the alpha, the closer to lasso (alpha=1), and the fewer variables are chosen. If the users select Net, they can include clinical variables in grouped variable selection.

In the user service, users can perform univariate or grouped variable selections after uploading their own data. Instructions on uploading data are detailed in the manual. Once data are uploaded, all the abovementioned analyses can be performed.

Finally, when developing gene signatures using selected variables, users should choose the cancer type, genes of interest, and time for the ROC curve, in that order.

### Univariate Analysis: Single Gene Analysis in Pan-Cancer

**Figure 2:**
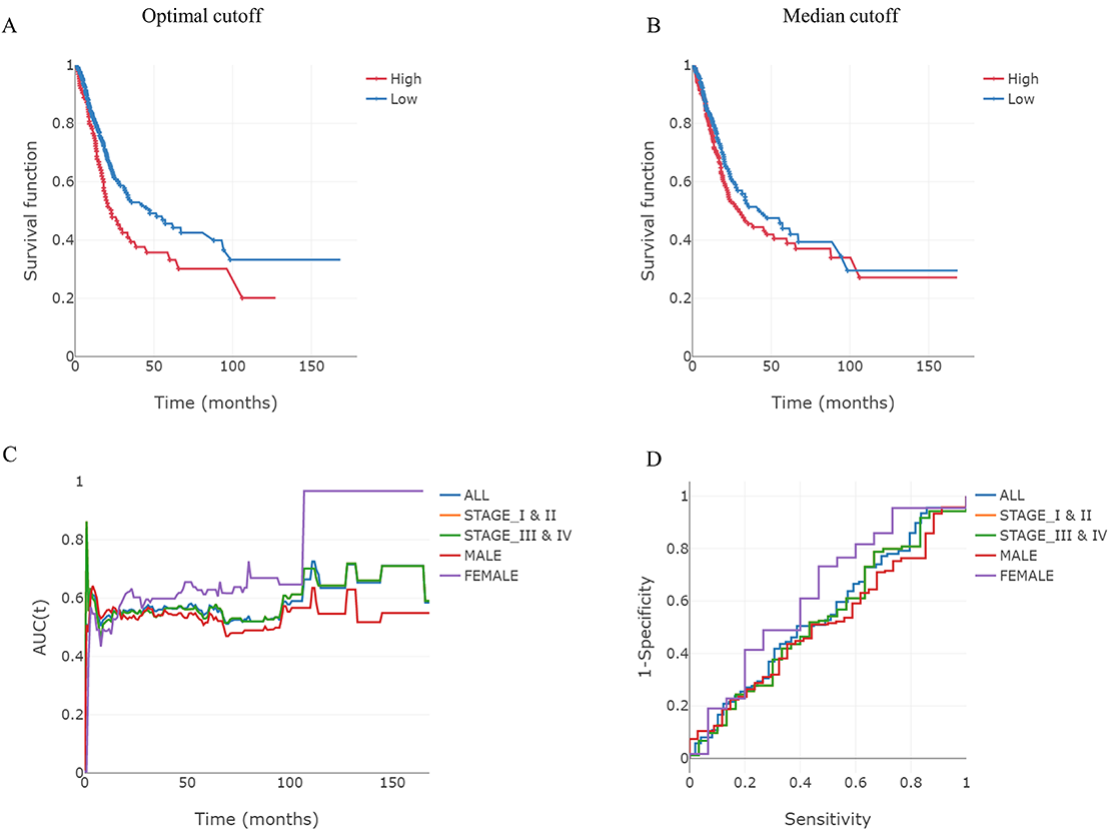
An example of mRNA-based survival analysis. Expression levels of genes are classified as low or high (blue or red lines, respectively) based on the comparison of their optimal (A) and median cut-off values (B). (C) Time-dependent area under the curve (AUC) for each of these subgroups. (D) Receiver operating characteristics (ROC) curves for selected years in each of these subgroups.

ESurv determines the prognostic significance of single genes as categorical (Kaplan-Meier curve with median or optimal cutoff values) or continuous variables (C-index and AUC values at specific time points) in various subgroups including sex and cancer stage (early and late as defined by the American Joint Committee on Cancer). To complement the results from the categorical variables, the C-index and AUC values were calculated as continuous variables. For example, we performed survival analysis using mRNA (*SLC2A1*) as a biomarker in bladder urothelial carcinoma (n=403; Figure 2). As shown in Figure 2A and B, the discrimination power with optimal cutoff values is much better than that of analyses completed using median cutoff values in all patients. Users easily obtain survival curves in subgroups as well as for all patients. In the time-dependent ROC curve analyses, users can identify the AUC value based on the follow-up time (Figure 2C). ROC curves at selected times can all be calculated (Figure 2D).

**Figure 3:**
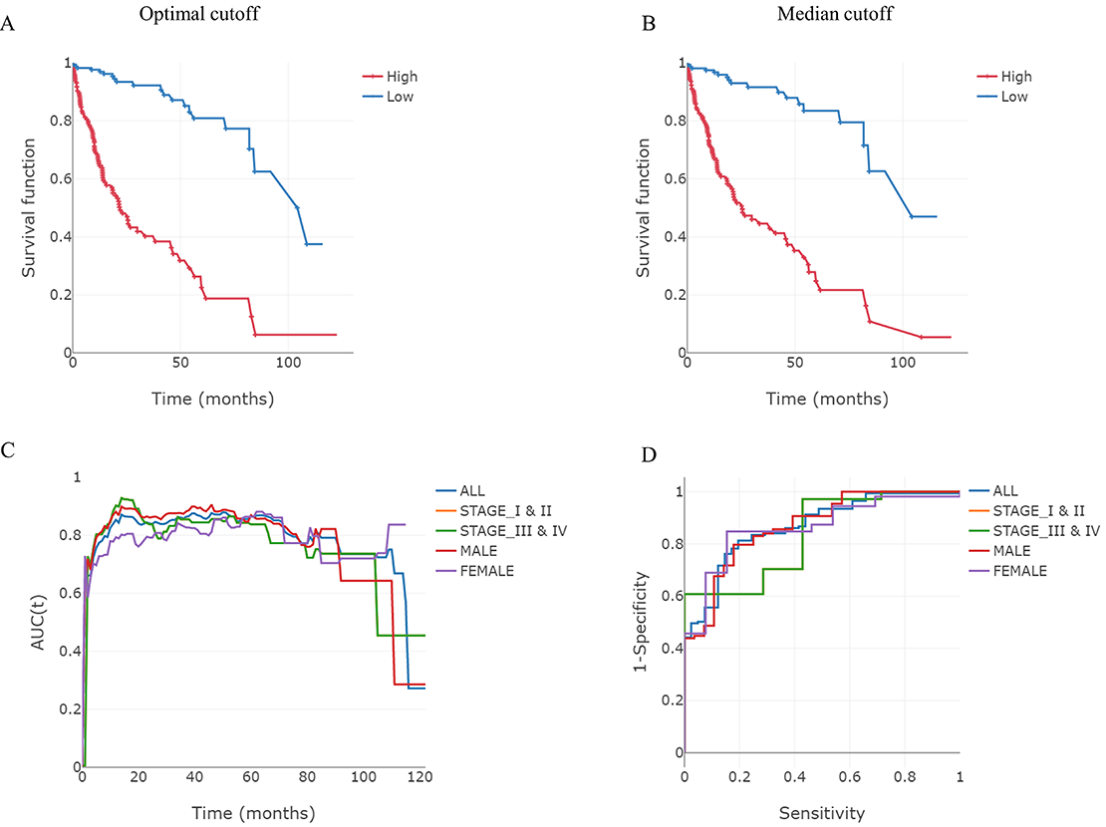
An example of survival analysis using a variable signature. Expression levels of genes were classified as either low or high (blue or red lines, respectively) based on a comparison of their optimal (A) and median cut-off values (B). (C) Time-dependent area under the curve (AUC) for each of the subgroups. (D) Receiver operating characteristics (ROC) curves for selected years in each of these subgroups.

### Univariate Analysis: Log-Rank Test of Whole Genomes

If the users want to calculate the prognostic value of all gene variants in a particular cancer, they can choose to do a log-rank test for the whole genome. Here, we performed a log-rank test of whole genomes in colon adenocarcinoma with a *P*<.05. ESurv provided the results (gene name, C-index, AUC value at user-selected time, *P* value of log-rank test) as an Excel file (Multimedia Appendix 1).

### Grouped Variable Selections

Grouped variable selection methods have been developed to take advantage of advances in biological technology, and can be used in statistical models to accurately predict the prognosis for a patient [2,10]. To select a prognostic gene set from high-dimensional data, it is necessary to select and minimize the number of variables in a systematic and statistically sound manner. ESurv provides representative grouped variable selection methods (lasso, elastic net, and Net), which can reduce the number of variables by considering the relationships between them. To develop a risk-scoring system, we used a linear combination of the expression values and regression coefficients of the selected variables. Users can develop a powerful prognostic signature in pan-cancer using any combination of these options. To illustrate this, we performed Net using all the clinical variables in hepatocellular carcinoma (n=307; Figure 3). Selected variables and regression coefficients can be downloaded from the summary tab in ESurv as Excel files. The results for all possible subgroups were recorded, just as in the case of single gene analysis. Outputs for the analysis can be in the form of Kaplan-Meier curves, time-dependent ROC curves, and AUC values at specific time points (Figure 3).

### User Service

Users can conduct univariate analysis, log-rank tests, and grouped variable selections by uploading their own data (Figure 1C). The manual is provided in Multimedia Appendices 2 and 3. Users can generate all survival results, as described above, using their own data sets. To protect data integrity, user-derived data sets are password-protected and connected to a unique user ID. In addition, as users refine their needs, they can request additional survival analysis packages via email. The most-requested package will be added to ESurv the following year.

## Discussion

### Principal Findings

To overcome the limitations of existing survival analysis tools, we developed a web-based user-friendly tool called ESurv. Kaplan-Meier curves are a common method of conducting survival analysis in the medical field, which involves categorizing patients into groups based on their risk profile. There is no clear criterion to classify continuous variables, like gene expression, in these categorical analysis methods. For this reason, median and quartile cutoff values used in previous tools may miss the prognostic significance of individual genes. As shown in Figure 2B, the median cutoff is not a suitable parameter for risk stratification, whereas optimal cutoff is a good parameter for risk stratification. Additionally, survival analysis as a continuous variable should be accompanied by these results to evaluate the prognostic significance of the gene of interest. Researchers can identify the prognostic significance of a gene (mRNA, miRNA, and methylation) as both a categorical and continuous variable in ESurv.

Prognosis using multiple genes yields superior results compared to using a single gene. There are a number of ways to select gene signatures, but among these, genes selected using grouped variable selection have proven to be the most versatile and reproducible [2,5,9,10,13,29,30]. Grouped variable selection methods select and shrink variables from high-dimensional data sets while considering multicollinearity, which is especially valuable when considering biological pathways, making Net options ideal for these applications. Despite this, grouped variable selection has not been applied to many studies because of the difficulty surrounding its computer programming (R, Python, and Matlab). To address this gap, we added grouped variable selection methods (lasso, elastic net, and Net) to ESurv. When selecting a prognostic gene set from high-dimensional data, researchers have to minimize the number of variables. In order to reduce variables effectively, we must provide as much information as possible linking genes and their relevant pathways [2,10]. It is easier to complete external validation with Net than with other methods because it performs variable selection using information about each gene, derived from databases hosting information pertaining to genetic pathways (Reactome, HumanCyc, KEGG, Biocarta, NCI, and Panther) [2,10,29,30]. Like with the univariate analyses, users can obtain the results as continuous and categorical data. ESurv is the first web-based tool to provide grouped variable selection using multiomics data.

The prognosis of patients may vary based on clinical information, such as sex and stage [9,31-35]. For these reasons, subgroup analysis is required to identify stage- and sex-specific prognostic genes. ESurv shows the results of survival analyses by taking into consideration tumor heterogeneity based on several classifications (cancer type, stages, and sex).

### Limitations and Future Work

This software does have some limitations that will be addressed upon further development. Here we used only one cancer database but there are many more, which we plan to add to ESurv as we continue to develop the software. Although users can upload their own data, this still requires users to be computer savvy; this will be addressed in future versions of the software. In addition, ESurv currently only accesses cancer databases, but this type of analysis is valuable in other diseases, including several vascular and degenerative diseases; we aim to add these as well. Finally, ESurv is not exhaustive in its analyses, but it is possible for users to request additional survival analysis packages for R via email. We will then select the most requested package and add it to ESurv on an annual basis.

### Conclusions

The most important functionality provided by ESurv is that users can use this software to analyze their own data. As more medical data is produced, the demand for survival analyses increases. Analysis of data created by individual institutions is as important as big data analysis, but there have been no survival analysis tools available to conduct this type of analysis. Using advanced statistical methods and comprehensive survival analyses, ESurv overcomes the limitations of previous tools, and allows users to work on their own data sets. We strongly believe that ESurv is an ideal tool to meet the growing demand for increased survival analysis in both small and large data sets.

## Appendix

Multimedia Appendix 1 The univariate results of whole significant genes.

Multimedia Appendix 2 The manual of univariate analyses.

Multimedia Appendix 3 The manual of grouped variable selection analyses.

## Abbreviations

AUC: area under the curve
Elastic net: elastic net regularization
ESurv: Easy, Effective, and Excellent Survival analysis tool
Lasso: least absolute shrinkage and selection operator
miRNA: microRNA
mRNA: messenger RNA
Net: network-regularized high-dimensional Cox-regression
ROC: receiver operating characteristics
TCGA: The Cancer Genome Atlas
