# Supplementary material for: A User-Friendly, Web-Based Integrative Tool (ESurv) for Survival Analysis: Development and Validation Study
Source: J Med Internet Res. 2020 May 5;22(5):e16084. doi: 10.2196/16084 (PMC7238095; doi:10.2196/16084)
Supplement: Multimedia Appendix 3 [file jmir_v22i5e16084_app3.docx]

ESurv manual – grouped variable selection analyses

1. Clinical data format

File name: clinical.RData

Object structure: data.frame

Object name: clinical

Example (clinical)


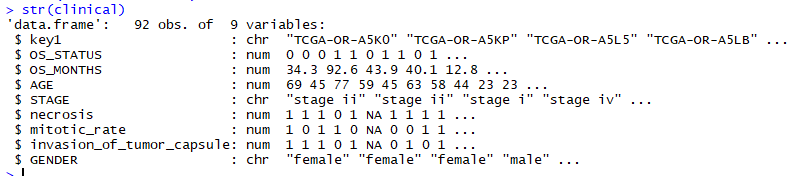


###Be sure to distinguish between uppercase and lowercase letters###

● Required input: key1, OS_STATUS, OS_MONTHS

→ The above fields must be input for survival analysis.

→ The variable name and type must be set as shown in the example above.

→ key1: should consist of patient IDs that match the gene data

→ OS_STATUS (overall survival status): deceased (1), alive (0)

→ OS_MONTHS (overall survival months): survival time

● Selective input: AGE, GENDER, STAGE, etc.

→ The above fields are optional for survival analysis.

→ The variable name and type must be set as shown in the example above.

→ GENDER: MALE/male/FEMALE/female

→ STAGE: Stage I / Stage IA / Stage IB / Stage IC / Stage IS / Stage II / Stage IIA / Stage IIB / Stage IIC / stage i / stage ia / stage ib / stage ic / stage is / stage ii / stage iia / stage iib / stage iic / Stage III / Stage IIIA / Stage IIIB / Stage IIIC / Stage IIIC1 / Stage IIIC2 / Stage IV / Stage IVA / Stage IVB / Stage IVC / stage iii / stage iiia / stage iiib / stage iiic / stage iiic1 / stage iiic2 / stage iv / stage iva / stage ivb / stage ivc

→ Other clinical variables can be entered as desired.

→ Numeric variables can be input as they are, and character variables must be transformed into 0 or 1.

2. Gene data format

File name: mRNA.RData or miRNA.RData or methylation.RData

Object structure: data.frame

Object name: mRNA or miRNA or methylation

Example (mRNA)


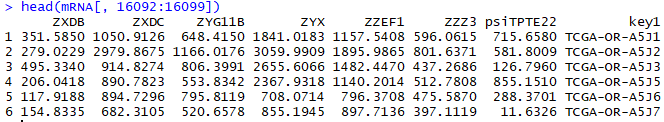


● Required input: key1, gene

→ key1: should consist of patient IDs that match the clinical data.

→ gene data: the gene names and expression values of each patient should be entered.

Example (miRNA)


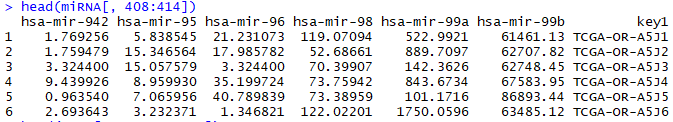


● Required input: key1, gene

→ key1: should consist of patient IDs that match the clinical data.

→ gene data: the gene names and expression values of each patient should be entered.

Example (methylation)


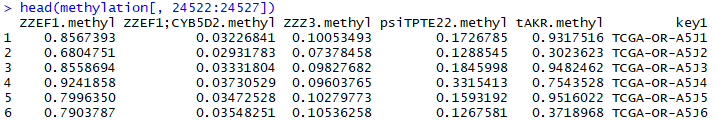


● Required input: key1, gene

→ key1: should consist of patient IDs that match the clinical data.

→ gene data: the gene names and expression values of each patient should be entered.
